# High-throughput sequencing of nodal marginal zone lymphomas identifies recurrent *BRAF* mutations

**DOI:** 10.1038/s41375-018-0082-4

**Published:** 2018-02-28

**Authors:** V. Pillonel, D. Juskevicius, C. K. Y. Ng, A. Bodmer, A. Zettl, D. Jucker, S. Dirnhofer, A. Tzankov

**Affiliations:** 1 Institute of Pathology and Medical Genetics, University Hospital Basel, University of Basel, Basel, Switzerland; 2 Department of Biomedicine, University Hospital Basel, Basel, Switzerland; 3 Institute of Pathology, Cantonal Hospital Baselland, Liestal, Switzerland; 4 Pathology, Viollier AG, Allschwil, Switzerland

## Abstract

Nodal marginal zone lymphoma (NMZL) is a rare small B-cell lymphoma lacking disease-defining phenotype and precise diagnostic markers. To better understand the mutational landscape of NMZL, particularly in comparison to other nodal small B-cell lymphomas, we performed whole-exome sequencing, targeted high-throughput sequencing, and array-comparative genomic hybridization on a retrospective series. Our study identified for the first time recurrent, diagnostically useful, and potentially therapeutically relevant *BRAF* mutations in NMZL. Sets of somatic mutations that could help to discriminate NMZL from other closely related small B-cell lymphomas were uncovered and tested on unclassifiable small B-cell lymphoma cases, in which clinical, morphological, and phenotypical features were equivocal. Application of targeted gene panel sequencing gave at many occasions valuable clues for more specific classification.

## Introduction

Nodal marginal zone B-cell lymphoma (NMZL) is a rare disease and represents <2% of all lymphoid neoplasms and approximately 10% of marginal zone B-cell lymphomas (MZL). Among MZL, which also comprise splenic MZL (SMZL) and extranodal MZL (EMZL) [1–3], it is the least studied entity, and has the least favorable prognosis [4, 5]. NMZL is a diagnosis of exclusion defined by the current classification of Tumors of Hematopoietic and Lymphoid Tissues of the World Health Organization (WHO) as “primary nodal B-cell neoplasm that morphologically resembles lymph node involvement by MZL of extranodal or splenic types, but without evidence of extranodal or splenic disease” [2]. NMZL has no disease-defining phenotype and its diagnostic borders to other small B-cell lymphomas such as EMZL, SMZL, and, particularly, lympho-plasmacytic lymphomas (LPL) are blurred [3, 6].

Previous array-comparative genomic hybridization (aCGH) studies of NMZL have identified recurrent gains of chromosomes 3, 12, and 18, but individual genes related to oncogenesis have not been further defined [6–9]. Recurrent chromosomal translocations that are frequent in other lymphoid malignancies are absent in NMZL [9]. A recent whole-exome sequencing (WES) study identified oncogenic mutations in genes involved in NOTCH, nuclear factor-κB (NF-κB), B-cell receptor, and toll-like receptor signaling pathways [10]. Some mutations have been proposed as diagnostic genetic biomarkers (*PTPRD*) or as prognostic biomarkers (*KLF2* and *NOTCH2*) for NMZL [9], but need validation for their clinical utility. Another study using targeted high-throughput sequencing (HTS) has shown frequent mutations of *TNFAIP3* encoding for the NF-κB signaling suppressor A20 [11].

Given the paucity of molecular data in NMZL, especially the lack of evidence for oncogenic pathways to which they may be “addicted”, targeted therapeutic approaches, other than application of anti-CD20 monoclonal antibodies, have not been applied. In general, NMZLs are treated in a similar manner to follicular lymphomas (FL) [12], but relapse more frequently [4] and are more aggressive in comparison to the latter [1]. Thus, there is an unmet need to better understand the molecular basis of NMZL. First, it would help to better define the diagnostic borders between NMZL and other closely related entities like “Bcl2-negative” FL, LPL, MZL of mucosa-associated lymphoid tissue (MALT), SMZL (in all of them recurrent mutations have already been identified [2]) as well as between NMZL and unclassifiable small B-cell lymphomas (SBCL, U) [2]. Second, detection of (recurrent) mutations in genes encoding for targetable molecular pathways may help to develop more tailored and more efficient therapies for NMZL.

In this study we used WES, targeted HTS, and aCGH to comprehensively characterize the genetic background of NMZL. We also sequenced cohorts of EMZL, SMZL, and LPL, and combined our genetic data with previously published data sets aiming to pinpoint differential genetic events between NMZL and these closely related entities. Finally, we applied our molecular data to a cohort of SBCL, U to test whether the most probable diagnostic category predicted by mutations fits with the integrative clinico-pathological entity.

## Material and methods

### Patients

The NMZL cohort consisted of 25 patients (8 discovery and 17 screening cases). All NMZL were BCL2 immunohistochemically (clones SP66 and E17) positive and t(14;18) negative, 18/22 (82%) were IgD-positive, 19/25 (76%) were BCL6-negative, and 6 were weakly (considerably weaker than preexisting germinal center B cells) BCL6-positive, while co-expressing IgD and being negative for *BCL6* rearrangements by fluorescence in situ hybridization (FISH; Suppl. Table 1). For comparative purposes, other small B-cell lymphomas were also included: 32 EMZL; 12 SMZL; and 11 LPL. In addition, 16 SBCL, U were analyzed aiming to leverage molecular genetic information for improving diagnostic classification (Suppl. Table 1); these cases consisted of small B-cell lymphomas without specific phenotype for which no absolute consensus on a final diagnostic entity could be reached, e.g., cases suggestive of nodal spread by LPL but without evidence of M-gradient, Waldenstöm macroglobulinemia, or bone marrow involvement, or cases suggestive of NMZL but with high M-gradients. The clinical, histopathological, and phenotypic characteristics of the series are summarized in Table 1 and Suppl. Table 1. Diagnoses were made according to the 2017 WHO classification [2]. Matched DNA from non-malignant tissue was obtained in 6 NMZL patients of the discovery cohort and used for WES in order to define somatic variants. Retrieval of tissue and data were performed according to regulations of the local institutional review boards and data safety laws. The study was approved by the Ethics Committee of North-Western and Central Switzerland (EKNZ 2014-252).

**Table 1:** Patient and disease characteristics of the study cohorts

	NMZL	SMZL	EMZL	LPL	SBCL, U
Sample size, *n*	25	12	32	11^a^	16
Gender					
Male, *n* (%)	11 (44)	8 (67)	17 (53)	8 (73)	6 (38)
Female, *n* (%)	14 (56)	4 (33)	15 (47)	3 (27)	10 (62)
Age in years: median (range)	66 (40–91)	68 (41–91)	64 (27–85)	71 (46–85)	74 (52–89)
Primary location: organ (*n*)	Lymph node (25)	Spleen (12)	Stomach (5); orbit (9); lung (6); skin (4); others (8)	Lymph node (6); bone marrow (5)	Lymph node (9); bone marrow (4); others (3)
Extranodal involvement					
All sites, yes/no (NA)	17/4 (4)	12/0 (0)	32/0 (0)	9/0 (2)	11/0 (5)
Bone marrow, yes/no (NA)	7/8 (10)	9/1 (2)	4/11 (17)	8/0 (3)	9/2 (5)
Splenomegaly, yes/no (NA)	10^b^/15 (0)	12/0 (0)	2/16 (14)	2/6 (3)	4^c^/4 (8)
M-gradient (paraprotein)					
Yes/no (NA)	1/10 (14)	3/5 (4)	6/8 (18)	4/2 (5)	2/4 (10)
Type (*n*)	IgG (1)	IgM (2), IgA (1)	IgM (6)	IgM (4)	IgM (1), IgG (1)
Stage, Ann-Arbor, *n*: I, II, III, IV (NA)	1, 5, 4, 5 (10)	1, 0, 0, 5 (6)	3, 1, 1, 3 (24)	0, 0, 0, 0 (11)	1, 0, 1, 0 (14)
Polyneuropathy, *n*: yes/no (NA)	0/12 (13)	1/7 (4)	3/13 (16)	1/2 (8)	1/6 (9)
Hemolytic anemia, *n*: yes/no (NA)	1/15 (9)	3/6 (3)	0/18 (14)	1/3 (7)	2/5 (9)
B-symptoms, *n*: yes/no (NA)	6/6 (13)	5/3 (4)	4/12 (16)	0/5 (6)	1/4 (11)
HCV, *n*: pos./neg. (NA)	0/6 (19)	1/7 (4)	0/7 (25)	0/3 (8)	0/4 (12)

*NMZL* nodal marginal zone lymphoma, *SMZL* splenic marginal zone lymphoma, *EMZL* extranodal marginal zone lymphoma, *LPL* lympho-plasmacytic lymphomas, *SBCL, U* small B-cell lymphoma unclassifiable, *LN* lymph node, *SP* spleen, *BM* bone marrow involvement, *IgM or IgG* immunoglobulin M or G paraprotein, *HCV* hepatitis C virus; *NA* not available

^a^ Eight of 11 LPL fulfilled criteria of Waldenström macroglobulinemia

^b^ One patient suffered from cardiomegaly

^c^ One patient suffered from hepatomegaly without splenomegaly

### Immunohistochemistry, FISH, and t(14;18) PCR/fragment length analysis

Immunohistochemistry and FISH were performed on serial tissue sections using an automated immunostainer Benchmark XT (Ventana/Roche, Tucson, AZ, USA) according to routine standard operation procedures and hybridization procedures as described elsewhere [13]; details on antibodies, retrieval conditions, and cutoffs as well as FISH probes used are given in Suppl. Table 2. t(14;18) has been sought for and excluded in the NMZL cohort by means of PCR and fragment analysis as described [14].

### DNA extraction

Formalin-fixed, paraffin-embedded (FFPE) and available fresh frozen (FF) tissue samples were retrieved. Genomic DNA was extracted from FF and FFPE tumor samples containing a tumor cell fraction of more than 50%, as estimated by morphological analysis and CD20 immunostaining. The DNA extraction was performed according to the standard procedures utilizing GeneRead^TM^ DNA-FFPE-Kit for FFPE samples and DNeasy Blood&Tissue-Kit for FF samples (both from Qiagen, Hilden, Germany). DNA was quantified using Qubit fluorometer (Invitrogen, Eugene, OR, USA). DNA integrity was assessed by multiplex PCR assay as described [15].

### Sanger sequencing

Sanger sequencing was performed on all NMZL samples in the discovery cohort prior to WES to exclude *MYD88* L265P mutant (potentially unrecognized LPL) cases using standard procedures [16].

### WES, variant detection, and filtering

Eight best-preserved *MYD88* L265P-negative NMZL and six matched non-tumor samples with sufficiently intact (amplicons of 400 bp amplifiable) [15] gDNA were selected for WES to identify novel somatic mutations. Sequencing library were created using Agilent SureSelect Human All Exon V6 (Agilent Technologies, Santa Clara, CA, USA) and sequencing was performed on the Illumina HiSeq platform (2 × 100 bp). Tumor samples were sequenced with mean target coverage of 100× and matched normal controls with 60×. WES library preparation and data generation were performed by CeGaT (Center for Genomics and Transcriptomics, Tuebingen, Germany). WES read and coverage statistics are summarized in Suppl. Table 3. WES data are deposited in NCBI SRA (accession number: SRP130154).

Sequence reads in both tumor and germline samples were aligned to the reference human genome GRCh37 using Burrows-Wheeler Aligner (v0.7.12) [17]. Local realignment, duplicate removal, and base quality adjustment were performed using the Genome Analysis Toolkit (GATK, v3.6) [18] and Picard (http://broadinstitute.github.io/picard/). Somatic single-nucleotide variants (SNV) were defined using MuTect (v1.1.4) [19]. Small insertions and deletions (InDels) were detected using Strelka (v2.0.15) [20].

Variants were annotated with Annovar and dbNSFP v3.0. For tumors with matched germline, the germline was used as control. For tumors without matched germline, an unmatched germline pool was created from the six available germline samples, subsampled to 16% (~1/6) of the reads and used as control. Hotspot variants [21] were white-listed. SNVs and InDels outside of the target regions, those with variant allelic fraction (VAF) of <5%, and/or those supported by <10 reads and/or those with <5× depth in the normal samples, and/or with <10× depth in the tumor samples were filtered out. We further excluded SNV and InDels for which the VAF in a tumor sample was <5 times that of the VAF in the matched normal sample. Variants identified in at least two normal samples using the artifact detection mode of MuTect (implemented with GATK v3.6) were flagged [22]. In addition, in unpaired samples, mutations that had population frequency >0.0001 according to ExAC database in non-Finnish European population and those with VAF between 45–55% and >95% were excluded to reduce contamination with germline variants.

### Somatic mutational signatures

All somatic point mutations detected by WES in the six cases with paired non-tumoral DNA were used to determine mutational signatures as described by Alexandrov et al. [23] using R package deconstructSigs [24]. Normalization for trinucleotide counts in human exomes and COSMIC signature database were utilized.

### Targeted HTS with custom lymphoma panel and data analysis

We supplemented an existing customized IonTorrent AmpliSeq HTS lymphoma panel (Thermo Fisher Scientific, Carlsbad, CA, USA) for targeted sequencing [25] based on recurrent findings in the discovery exomes and added genes reported to be affected in MZL, including NMZL [10]. Further, we included regions that are most frequently mutated in B-cell lymphomas according to the COSMIC database (release v82) and the literature [26]. In the final design 146 target genes (32 all exons and 114 selected regions only) were included (Suppl. Table 4).

All 96 samples described in this study were sequenced by the IonTorrent S5XL instrument using the Ion540 sequencing chip with mean coverage depth of 1400× (range 748–2962×). Statistics regarding coverage depth, uniformity, and mapping quality are summarized in Suppl. Table 5. Targeted sequencing data were deposited in NCBI SRA (accession number SRP130154). Mutation identification was performed by the Variant caller plug-in v5.2 IonTorrent software suite (Thermo Fisher Scientific) using generic low stringency parameters for somatic mutations. Variants were annotated with the IonReporter software and dbNSFP database v3.0 [27] and filtered according to criteria listed in Suppl. Table 6. Positions of all remaining mutations were manually inspected in mapped BAM files using the IGV viewer in order to exclude any remaining artifacts. Functional annotations of somatic point mutations were performed by ensemble MetaLR score combining multiple functional prediction and conservation algorithms [27].

We ran a validation experiment on a small set of samples to determine whether sequencing results were consistent between DNA samples derived from FF and FFPE tissues, technical replicates (FFPE), and intratumoral replicates (FFPE). For validation samples were chosen randomly according to required material type. After quality filtering, somatic and germline variant calls in replicates were compared and concordance was calculated. In addition, we compared somatic mutation calls between WES and targeted HTS for genes included in our custom lymphoma panel.

### Meta-analysis of mutated gene frequencies

Gene mutation frequency data were retrieved from previously published sequencing studies with data accessibility for NMZL [8, 16, 28, 29], LPL [16, 29–31], EMZL [16, 32–34], and SMZL [16, 29, 35–40]. For FL, the COSMIC data set containing gene mutation frequencies for 407 samples was used [26]. Variant tables listing all identified somatic mutations were downloaded to calculate the number of mutated and unmutated cases gene-wise. These data were pooled within entities and used for inter-entity comparison (Suppl. Table 11). Number of mutated cases ≥4 and frequency of ≥5% was used as a cutoff to generate a gene shortlist, for which statistical differences between entities were calculated by the two-tailed Fisher’s exact test.

### Array-comparative genomic hybridization

aCGH (Agilent SurePrint 180k, mean resolution 13 kb) analysis (Agilent Technologies) was performed on 22 NMZL cases with sufficient DNA applying well-established techniques [25, 41, 42]. aCGH data were analyzed using Agilent Genomic Workbench v.7.0 software with the aberration detection algorithm ADM-2 (threshold 12.0) [43]. Copy number data are deposited in Gene Expression Omnibus (accession number GSE109487).

### Pathway analysis

Gene sets of manually curated canonical cell signaling pathways were downloaded from the Molecular Signature Database v6.0 (http://software.broadinstitute.org/gsea/msigdb) (Suppl. Table 7). Frequency of pathway deregulation was computed with a custom workflow programmed in R. A pathway was considered affected if at least one gene encoding a pathway component contained a non-synonymous genetic alteration.

### Statistical analysis

Two-tailed Fisher’s exact and Wilcoxon rank sum tests were utilized to determine the significance of mutational distribution differences between groups. All statistical calculations were performed with MS Excel, SPSS22, or R statistical package. Statistical significance threshold of *p* < 0.05 was assumed in all analyses.

## Results

### Study cohorts

All relevant clinico-pathological information of our study cohorts are detailed in Table 1 and Suppl. Table 1.

### Genes encoding for chromatin remodeling and transcriptional regulation are recurrently mutated in NMZL

**Fig. 1:**
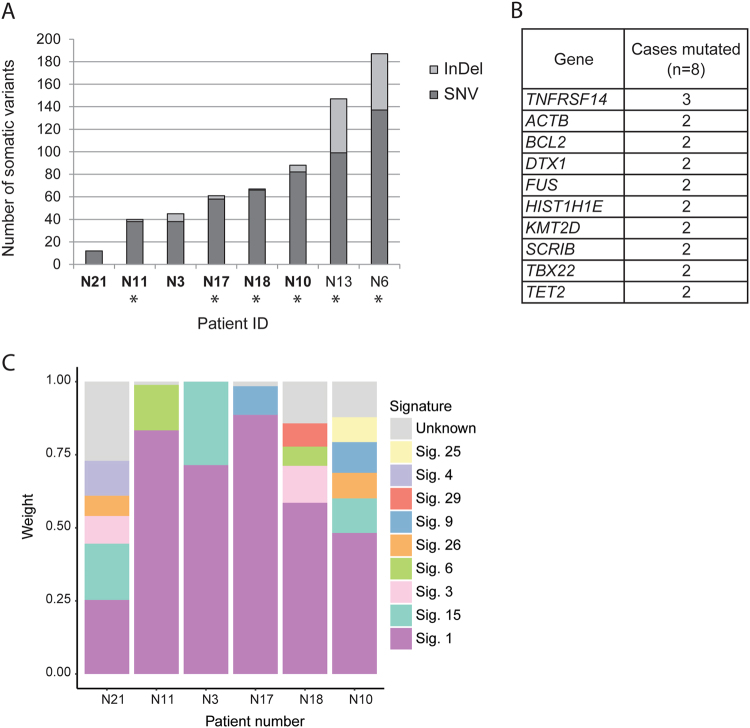
Whole-exome sequencing of NMZL. **a** Number and type of single-nucleotide variants (SNVs) and insertions/deletions (InDels) identified in the 8 discovery exomes. Asterisks indicate tumor DNA extracted from fresh frozen tissue. Tumor samples with matched germline tissue are in bold. **b** Recurrently mutated genes (≥2/8 cases mutated, and selected based on functional annotation and previous data in MZL) detected in the NMZL discovery cohort (*n* = 8) by whole-exome sequencing. **c** Mutational signatures based on synonymous and non-synonymous somatic point mutations identified in exomes of NMZL with available paired non-tumoral tissue

To characterize the genetic landscape of NMZL, we performed WES of 8 NMZL samples. For 6 samples matched non-tumoral DNA was also sequenced (Suppl. Table 3). We identified a total of 647 non-synonymous somatic mutations (313 in paired samples only), including 530 SNVs and 117 small InDels (294 SNVs and 19 InDels in paired samples only; Suppl. Table 8). The overall load of mutations/sample was heterogeneous across the 8 NMZL cases investigated, ranging from 12 to 187 (mean 81), and in the 6 paired cases, ranging from 12 to 88 (mean 52; Fig. 1a). We identified 43 candidate genes affected in ≥2/8 NMZL, and 16 genes affected in ≥2/6 NMZL in the paired samples. Among the most frequently affected genes were those encoding for chromatin remodeling and transcriptional regulation pathways: *KMT2D*; *TET2*; *HIST1H1E*; *FUS*; and *ACTB* (Fig. 1b). We used the set of somatic point mutations detected in the 6 paired tumor exomes to determine mutational signatures and confirmed that the aging-related mutational signature 1 characterized by an enrichment of C > T transitions in NpCpG context was the most predominant one (Fig. 1c), as reported previously in NMZL [10], and in other B-cell lymphomas [44–46]. Other frequently detected signatures were 9, 15, 3, 6, 26, and 25. Only signatures 9 and 25 have previously been associated with B-cell malignancies.

### Mutational load is higher in NMZL compared to EMZL

**Fig. 2:**
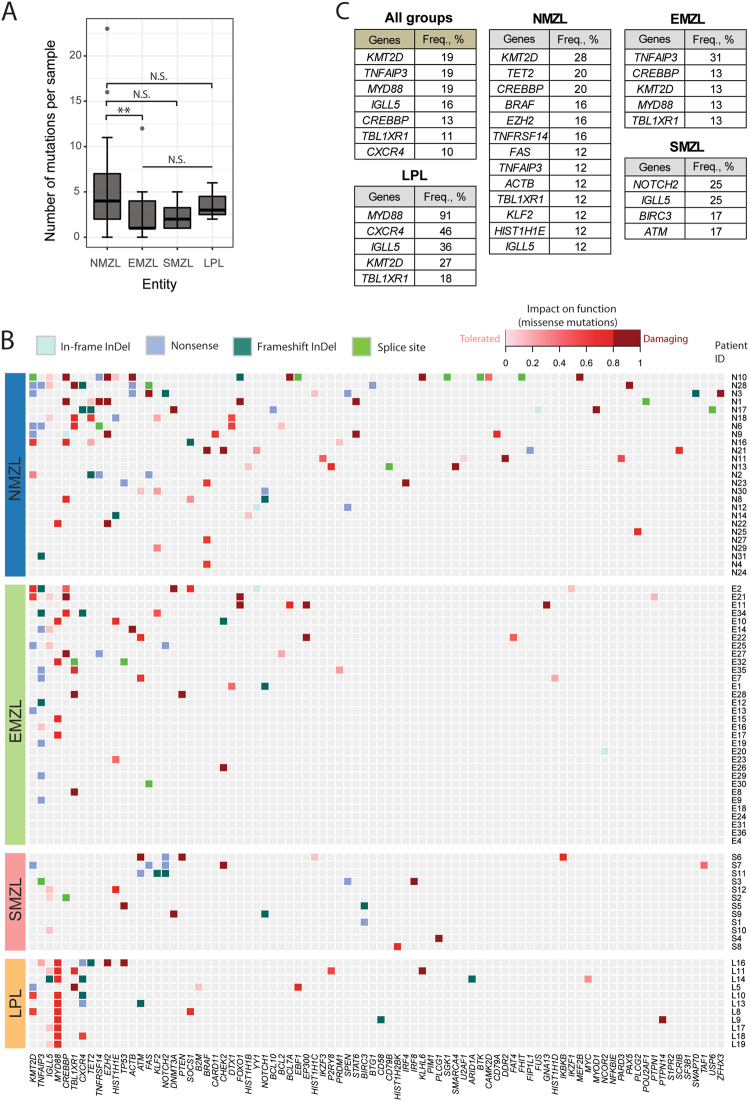
Landscape of somatic mutations in the studied small B-cell lymphomas. **a** Number of mutations per sample detected in the different cohorts. Statistical significance by Fisher’s exact test, **p < 0.01; NS not significant. **b** Heatmap plot showing all non-synonymous mutations detected by targeted high-throughput sequencing in the four small B-cell lymphoma cohorts (NMZL, *n* = 25; EMZL, *n* = 32; SMZL, *n* = 12; and LPL, *n* = 11). Each row represents a primary tumor grouped according to the assigned subtype. Each column represents a gene ordered left to right in decreasing order of detection frequency. When multiple mutations were present in the same gene, the most damaging mutation is displayed. **c** Most frequently mutated genes (frequency ≥10%), overall and in each studied entity

We performed targeted HTS on all samples from our four small B-cell lymphoma cohorts, i.e., NMZL, EMZL, SMZL, and LPL (Suppl. Table 9). In these 80 samples 281 mutations were detected in total and approximately 50% of them were disruptive for gene function (Suppl. Figure 1A). The most common substitutions in all cohorts were C > T/G > A and T > C/A > G transitions (Suppl. Figure 1B). The overall load of mutations/sample was significantly higher in NMZL compared to EMZL (*p* < 0.01, Wilcoxon rank sum test) and was heterogeneous across the 25 NMZL cases investigated, ranging from 0 to 23 mutations/case (mean 4; Fig. 2a). Validation of HTS data showed good concordance between technical, material type (FFPE or FF), and intratumoral replicates (Suppl. Figure 2A-C). Also there was a good concordance of 83% (60/72) between mutations detected by WES and targeted HTS (Suppl. Figure 2D and Suppl. Table 10).

### Recurrently mutated genes in NMZL

Of the 146 genes investigated by HTS, 84 were mutated at least once (Fig. 2b). *KMT2D* was the most frequently mutated gene in NMZL (7/25, 28%; Fig. 2c). Other epigentic modifiers *TET2* and *CREBBP* (both 20%, 5/25) were also frequently affected in NMZL, closely followed by *BRAF*, *EZH2*, and *TNFRSF14* (each 16%, 4/25). In EMZL the top mutated gene was *TNFAIP3*, while as expected LPL had most frequent mutations in *MYD88* and *CXCR4*. SMZL bore the most frequent mutations *NOTCH2* and *IGLL5* (both 25%). The latter gene was also found mutated in other entities and was the fourth most frequently mutated gene overall (Fig. 2c).

### *BRAF* mutations are recurrent in NMZL and may be of diagnostic and potential theranostic importance

**Fig. 3:**
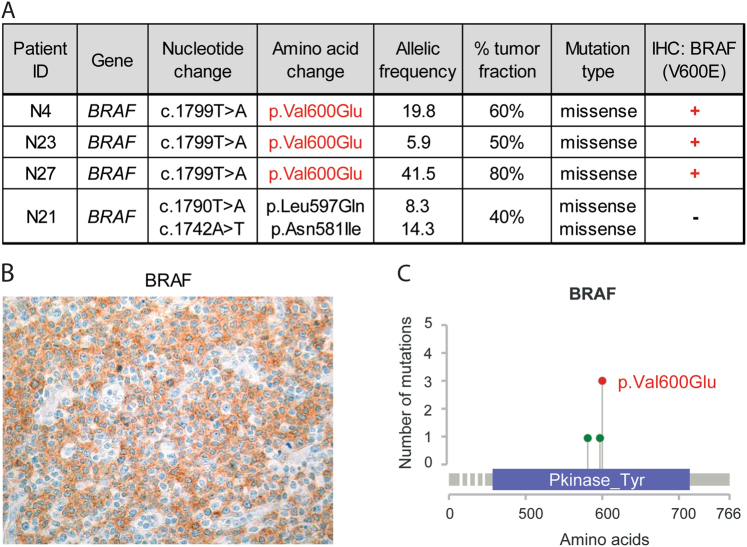
*BRAF* is recurrently mutated in NMZL but not in the other studied small B-cell lymphoma entities. **a** Details of *BRAF* mutations identified in NMZL. **b** Representative immunohistochemical staining of a lymph node biopsy of a *BRAF* mutant NMZL case with the anti-BRAF V600E (clone VE1) antibody. Note positively staining (moderate slightly granular cytoplasmic positivity) tumor cells and adequate internal negative controls. Original magnification: ×240. **c** Schematic representation of the protein tyrosine kinase domain (Pkinase_Tyr) of the human BRAF protein. Two of the detected non-hotspot mutations (in green) are located in the close proximity to the Val600 hotspot (in red) and are predicted to be damaging in silico

To our surprise we identified 4/25 *BRAF*-mutated NMZL cases (Fig. 3a). Three of them bore the canonical V600E hotspot mutation, also confirmed by immunohistochemistry with the validated [47] anti-BRAF-V600E antibody (Fig. 3b). The remaining case (N21) had two damaging mutations targeting amino acids close to the above hotspot—N581I and L597Q (Fig. 3c, Suppl. Table 9). Based on the tumor cell fraction and the variant allelic frequency we estimated that at least in cases N27 and N4 the *BRAF* mutations were clonal (VAF 42% and 20%, respectively). In order to exclude lymph node involvement by hairy cell leukemia (HCL), all *BRAF* mutant NMZL were carefully reevaluted and stained for CD103 and Annexin A1 and turned out to be negative; in addition, all of them were negative for cyclin D1. Importantly, all four cases (100%) were strongly positive for IgD, which was considerably more frequent than in *BRAF* wild-type cases, which expressed IgD strongly in 4/18 (22%) and weakly to moderately in 10/18 (56%) instances (*p* ≤ 0.01, Fisher’s exact test; Suppl. Table 1). No other common denominator respecting age, sex, stage, or outcome applied to these four cases.

### NMZL have recurrent lesions of genes involved in MZ B-cell development

**Fig. 4:**
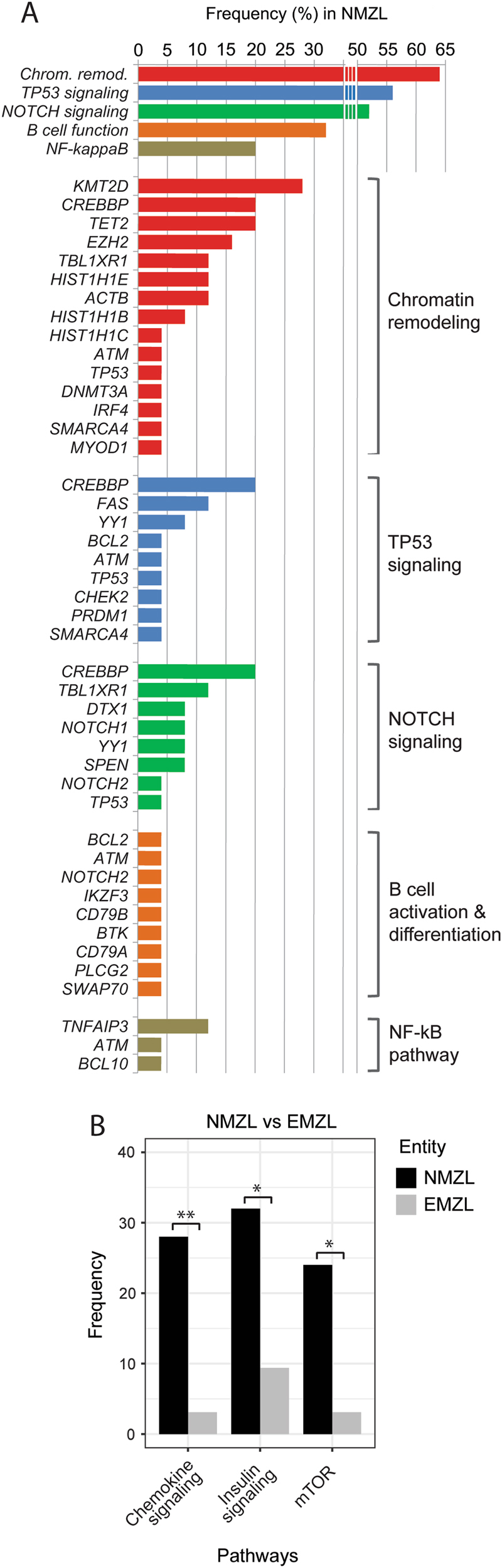
Recurrently mutated pathways. **a** Pathways that are recurrently affected by mutations in NMZL. The bar graphs represent the overall frequency of mutations in each pathway and the frequency of mutations in each gene grouped by pathway. **b** Differentially mutated pathways between NMZL and EMZL (Fisher’s exact test, *p < 0.05, **p < 0.01)

Overall, the genes that were recurrently mutated in NMZL are implicated in chromatin remodeling (64%), TP53 (56%), NOTCH (52%), and NF-κB- (20%) pathways as well as in B-cell activation and differentiation (32%; Fig. 4a). We further identified a significant enrichment of genes involved in chemokine-, insulin-, IL4-, and mTOR-signaling in NMZL compared to EMZL (Fig. 4b, Suppl. Figure 5).

### Trisomies of chromosomes 3, 12, and 18 are recurrent in NMZL

**Fig. 5:**
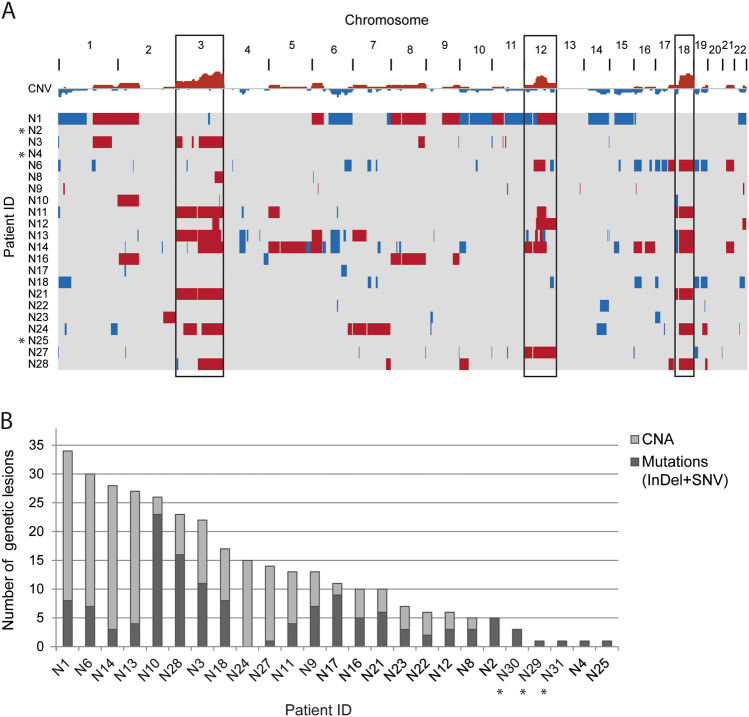
Copy number analysis of NMZL. **a** Array-comparative genomic hybridization (aCGH) of NMZL cases (*n* = 22). Red corresponds to gains, blue to losses, and gray to normal (diploid) copy numbers. Trisomies of chromosomes 3, 12, or 18 are highlighted in the boxes. Patients N2, N4, and N25 (indicated by asterisk) had no copy number aberrations (CNAs). **b** Combined load of somatically acquired genetic lesions identified in the NMZL screening cohort (*n* = 25), including the numbers of CNA and non-synonymous mutations. Patients samples for which aCGH was not performed are indicated by an asterisk

We performed aCGH of 22 NMZL with sufficient available DNA (Fig. 5a). On average there were nine copy number aberrations (CNA) per case (range 0–26). The most recurrent aberrations were trisomies of chromosomes 3, 12, and 18, confirming findings from previous studies [6, 8, 9, 28]. We observed mostly single copy gains and heterozygous losses, whereas high-level amplifications as well as homozygous deletions were absent. Three NMZL cases had no CNA. Notably, some NMZL cases, which had no or very few mutations detected by HTS, had higher than average CNA numbers (Fig. 5b). Sequencing and aCGH data combined, NZML had 13 somatic genetic lesions on average (range 1–34).

### Differential predominance of mutated genes in SBCL subtypes

**Fig. 6:**
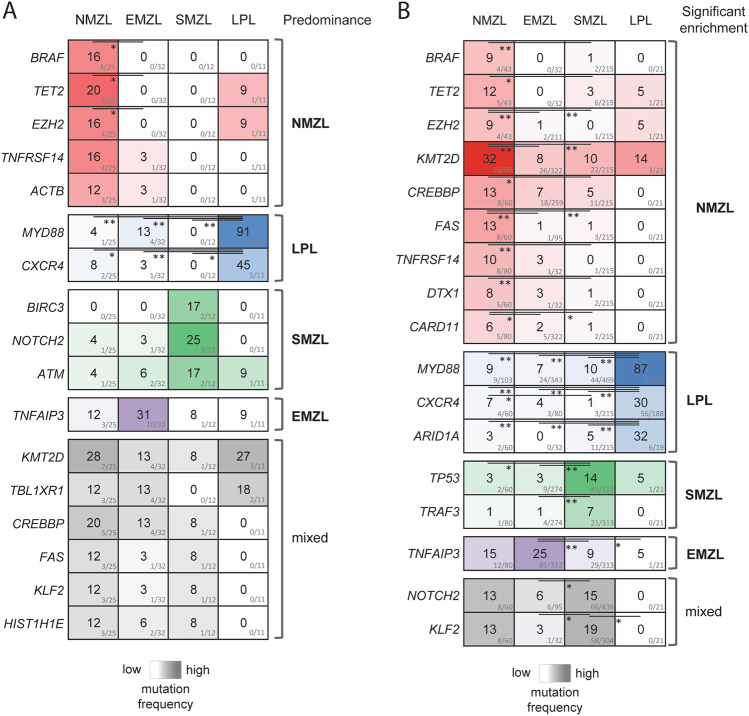
Differentially mutated genes between studied entities. **a** Genes that are differentially mutated in NMZL compared to EMZL, SMZL, and LPL; heatmap representing alteration frequencies in the differentially mutated genes grouped by predominance in the different subtypes. **b** Heatmap shows gene mutational frequency comparison results by meta-analysis, which included data from our current cohort and data retrieved from previously published sequencing studies [8, 16, 28–40]. Only statistically significant enrichments are shown, as determined by Fisher’s exact test, *p ≤ 0.05, **p ≤ 0.01

To assess whether some genes are predominantly mutated in one subtype of SBCL, we compared mutational frequencies within our study as well as by pooling our data with mutational data from previously published studies (Fig. 6a, b). Within our cohort, *BRAF* mutations were only found in NMZL and were statistically more frequent than in EMZL (*p* ≤ 0.05) and were numerically more frequent than in SMZL and LPL. In the meta-analysis of the pooled data set, *BRAF* mutations remained more frequent in NMZL (9%, 4/43) reaching statistical significance against SMZL (1%, 2/215, *p* ≤ 0.01, Fisher’s exact test). Similarly, meta-analysis confirmed a near exclusivity of *PTPRD* mutations to NMZL, however this difference did not reach statistical significance due to a small number of cases tested in all other entities (Suppl. Figure 4A). Further, NMZL displayed enrichment of mutations in the epigenetic modifiers *KMT2D*, *CREBBP*, *EZH2*, and *TET2*, which clearly separated NMZL from other SBCL entities, especially from SMZL and EMZL (*p* ≤ 0.05, Fisher’s exact test). Finally, mutations in *FAS* and *TNFRSF14*, both encoding important cellular receptors, were consistently found in NMZL by us and by Spina et al. [10], but were virtually absent in EMZL, SMZL, and LPL (*p* ≤ 0.01, Fisher’s exact test).

In agreement with previous findings [48] our cohort showed the predominant occurrence of *MYD88* and *CXCR4* mutations in LPL (Fig. 6a). Meta-analysis confirmed this result and, in addition, identified *ARID1A* as a third recurrently mutated gene in this neoplasm (Fig. 6b).

More frequent mutations in *TP53* and *TRAF3* differentiated SMZL from other SBCL. *NOTCH2* and *KLF2* mutations were previously found to be characteristic for SMZL [39, 40]. In our meta-analysis they occurred in 15% and 19% of SMZL cases, respectively, but both were also present in 13% of NMZL. Thus, mutations of *NOTCH2* and *KLF2* had a mixed predominance for these two entities.

In our cohort *TNFAIP3* was the most frequently mutated gene in EMZL (31%) as it was in the meta-analysis, which showed that *TNFAIP3* was mutated in 25% of EMZL cases (Fig. 6a, b), which was significantly more common in comparison to SMZL and LPL (*p* ≤ 0.01 and ≤0.05, respectively, Fisher’s exact test), but not to NMZL, where *TNFAIP3* was mutated in 15% of studied cases. Interestingly in five EMZL cases *TNFAIP3* mutations was the only found mutation (Suppl. Figure 3).

Since t(14;18)-negative FL represents an important differential diagnostic entity for NMZL, we also performed a meta-analysis of gene mutation frequencies between NMZL and FL. Interestingly, though predominant in NMZL compared to other SBCL, mutations in the epigenetic modifiers *KMT2D*, *EZH2*, and *CREBBP* were considerably more frequent in FL (Suppl. Figure 4B). Further, FL displayed recurrent *TP53* mutations occurring in 18% of cases, whereas this gene was affected only in 3% of NMZL (*p* ≤ 0.01, Fisher’s exact test). On the other hand, FL rarely had mutations of *TNFAIP3*, *KLF2, NOTCH2*, and *BRAF* (*p* ≤ 0.05, Fisher’s exact test); therefore, detection of aberrations in these genes could be useful in the respective differential diagnosis.

### Mutational data may be useful for further classification of ambiguous cases

**Fig. 7:**
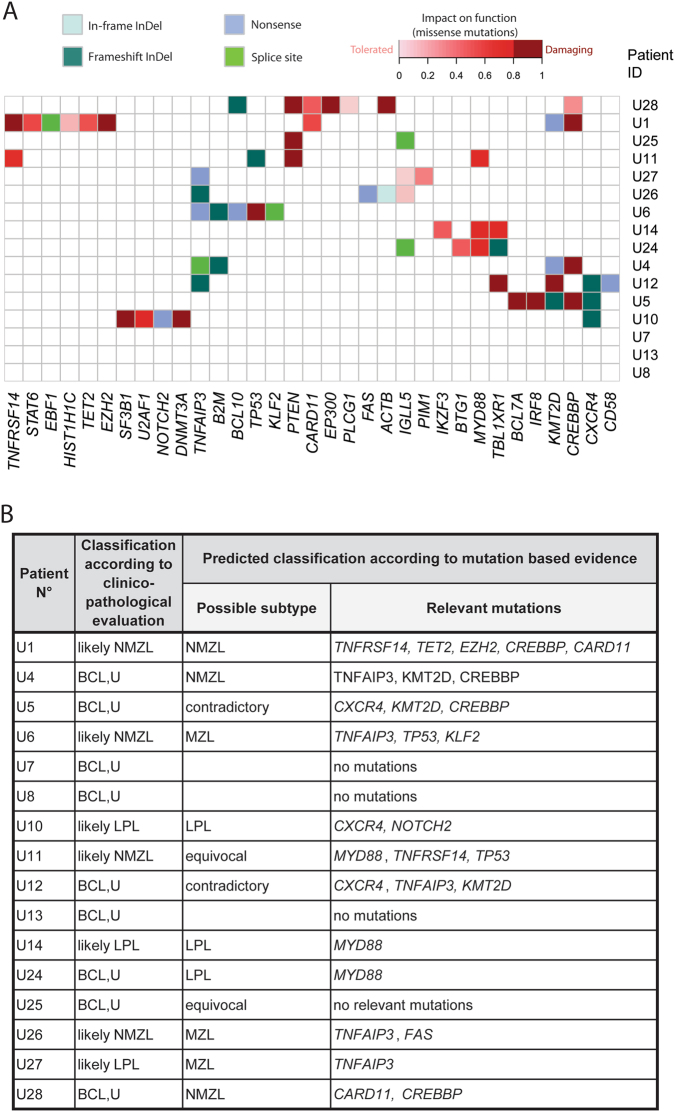
Mutational hints for the classification of unclassifiable small B-cell lymphoma cases. **a** Heatmap plot showing all non-synonymous mutations detected by targeted high-throughput sequencing in unclassifiable small B-cell lymphomas cases (SBCL, U). The heatmap is clustered by genes and samples with each row representing a primary SBCL, U tumor and each column representing a gene. **b** Clinico-pathological classification and predicted classification according to the mutation-based evidence of SBCL, U cases

We performed targeted HTS on 16 ambiguous cases falling in the category of SBCL, U (Suppl. Table1). Our aim was to investigate if mutational information would provide a valuable addition to routinely collected clinico-pathological data and help to better classify such cases into distinct diagnostic categories. We identified a total of 73 nonsilent somatic mutations, including 59 SNVs and 14 InDels (Suppl. Table 9). The overall load of mutations/sample was heterogeneous across the 16 SBCL, U cases, ranging from no mutation in 3 cases up to 11 lesions in 1 case (mean 5.6; Fig. 7a). In 12 cases we identified potentially diagnostically useful mutations in *CXCR4*, *EZH2*, *FAS*, *MYD88*, *TET2*, *TNFAIP3*, *KMT2D*, *CREBBP*, *KLF2*, *NOTCH2*, *TP53*, and *TNFRSF14* (Fig. 7b), as these occurred more frequently in one particular subtype of SBCL (Fig. 6b). In 5/16 cases (31%) mutational profile was in agreement with the clinico-pathological data, allowing to allocate the respective case to a most probable entity (Fig. 7b). In 2 cases, U14 and U24, a single relevant *MYD88* L265P mutation indicated likely LPL classification. Co-occurring *TNFAIP3*, *KMT2D*, and *CREBBP* mutations indicated a likely NMLZ in case U4. Same classification was concluded for cases U1 and U28 based on mutations in *CREBBP*, *CARD11*,* EZH2*, *TNFRSF14*, and *TET2*. Three cases (U5, U12, and U11) bore mutations that suggested contradicting classifications according to our current knowledge (*CXCR4* or *MYD88* indicative of LPL co-ocuring with *TNFAIP3*, *KMT2D*, *CREBBP*, *KLF2*, and *TP53* all indicative of MZL), and were therefore not conclusive. Reclassification based on mutational data could not be considered in 4 cases, where no somatic mutations or no mutations in genes with known entity predominance were detected.

## Discussion

In this study we aimed to comprehensively characterize the genetic background of NMZL using WES, targeted HTS, and aCGH, with a special emphasis on sharpening the diagnostic borders between NMZL and other closely related SBCL entities such as EMZL, SMZL, LPL, and BCL, U. Importantly, to rule out FL (mimicking NMZL), only cases proven to be t(14;18)-negative and BCL2-positive, and, in case of weak BCL6-positivity, without *BCL6* rearrangement, were included in the NMZL cohort.

The most remarkable result of our study is the discovery of recurrent hotspot *BRAF* mutations in 16% of NMZL. In our cohort they were exclusive to NMZL and occurred in cases with strong IgD expression. Until now, it was thought that among B-cell neoplasms *BRAF* V600E mutation is almost entirely restricted to HCL [2, 49–53], though individual *BRAF* V600E-mutant cases of chronic lymphocytic leukemia, prolymphocytic leukemia [54], classical Hodgkin lymphoma [55], and SMZL [56, 57] were also documented. Notably, no *BRAF* mutations have been detected in any other previous study on NMZL [10, 58]. *BRAF* encodes for a serine/threonine kinase that is commonly activated by a hotspot V600E somatic point mutation [59] and represents one of the most frequently mutated genes in cancer [26]. This hotspot kinase-activating mutation is a well-known therapeutic target for the BRAF kinase inhibitors [60, 61]. Hence, mutant *BRAF* may open new targeted therapeutic opportunities for NMZL, at least in cases in which this mutation is clonal. In addition, this finding has immediate practical implications for the diagnosis and differential diagnosis of NMZL, since the V600E mutation can be reliably identified by immunohistochemistry. The two non-hotspot mutations detected in one NMZL case, L597Q and N581I, were previously found in *BRAF* V600 wild-type melanoma and their part in oncogenic cell transformation was functionally proven [62, 63] thus further supporting the role of mutant *BRAF* in NMZL development. Interestingly, during the preparation of this manuscript we identified an additional *BRAF* mutant NMZL case in consultation: a CD45+/CD138+/MUM1+/CD19−/CD20−/CD56−/CD79a−/Cyclin D1− plasmacytoid tumor without myeloma-characteristic translocations and polysomies that was BRAF V600E+ by immunohistochemistry and displayed the V600E mutation by HTS (data not shown).

*TET2*, *EZH2*, and *BRAF* were among the more frequently mutated genes in NMZL in our study, but were not found mutated by other studies. The opposite was true for *PTPRD*, which was frequently mutated in the study of Spina et al. [10] but we found no mutations in our NMZL cohort. To a large extent such discrepancies may be explained by small sample sizes and thus the lack of statistical power to detect relatively rare mutations. In addition, due to the length of *PTPRD* gene and the absence of a clear mutational hotspot, we sequenced only those regions that were previously found mutated [10]. This means that mutations in the uncovered parts of the gene could have been overlooked. However, we did not detect any *PTPRD* mutation in the eight NMZL cases investigated by WES. On the other hand, consistent with previous genetic studies [10, 11] on NMZL, we detected *KMT2D* as the the most frequently mutated gene in this disease, as well as recurrent mutations in *CREBBP*,* TNFRSF14*, *FAS*, *TNFAIP3*, *KLF2*, and *CXCR4*. Moreover, our aCGH data also confirmed previous findings [6, 8, 9, 28] showing recurrent trisomies of chromosomes 3, 12, and 18.

We investigated specifically affected pathways and found that NMZL have recurrent lesions of genes involved in MZ B-cell development: among them, *NOTCH1*, *SPEN*, and *DTX1* of the NOTCH signaling pathway are known to be relevant for normal MZ differentiation [64–67]. Mutations of NF-κB pathway members were also recurrent in NMZL and affected several previously identified genes, including *TNFAIP3* and *BCL10* [10, 11]. Apart from their general importance for understanding the pathogenesis of NMZL and SMZL, such molecular pathways may point toward potential targets to develop more tailored and more efficient therapies for NMZL. Pharmacologic interference of NOTCH or NF-κB signaling could be an attractive approach in NMZL and SMZL, as already suggested by others [9]. Furthermore, in our NMZL collective recurrent mutations in genes encoding for chromatin remodeling and transcriptional regulation were detectable, including the histone methyltransferase *KMT2D*, the acetyltransferases *CREBBP* and *TBL1XR1*, an integral component of the N-Cor co-repressor complex. Such mutations in epigenetic regulators were previously reported to be present in approximately 40% of NMZL [10] and may provide therapeutic opportunities using agents such as histone deacetylase (HDAC) inhibitors. For example *CREBBP* mutant DLBCL was shown to be sensitive toward HDAC inhibitors in laboratory conditions [68].

Meta-analysis of mutational frequencies in SBCL showed that *BRAF*, *TET2*, *EZH2*, *KMT2D*, *CREBBP*, *TNFRSF14*, and *FAS* aberrations were almost exclusive to NMZL and might therefore be helpful to discriminate them from other closely related SBCL. Previously, *FAS* mutations were reported to be absent in MZL [69]. However, we and, earlier, Spina et al. [10] found *FAS* mutations in NMZL that were all disruptive to protein function. *FAS* encodes a receptor of tumor necrosis factor family, which plays a key role in extracellular apoptotic signaling [70]. Mutations, mostly occurring in proteins’ death domain, abolish its function and render cells resistant to apoptosis. Besides NMZL, *FAS* is frequently mutated in in patients suffering from the autoimmune lymphoproliferative syndrome [71], in adult T-cell lymphoma [72], and diffuse large B-cell lymphoma [73]. It can also be rarely mutated in gastric MZL of MALT [74] and cutaneous EMZL [75]. Indeed, the single EMZL case with *FAS* mutation in our study (E30) was of cutaneous origin. The pooled data analysis further clearly confirmed the predominant occurrence of *MYD88* L265P and *CXCR4* mutations in LPL. Notably, co-occurring *CXCR4* and *MYD88* mutations were exclusively found in LPL. Knowledge on these alterations can help diagnose LPL presenting outside of the typical clinico-pathological context (e.g., without an IgM M-gradient or bone marrow involvement) that might be otherwise misclassified as NMZL [2, 16, 76]. Additionally, our analysis identified a predominance of *ARID1A* mutations in LPL. The product of this gene is involved in chromatin remodeling [77]. However, even in the meta-analysis, the number of investigated LPL cases for *ARID1A* mutations was too small to allow drawing robust conclusions, therefore this finding remains to be further validated. In our study as well as in the meta-analysis EMZL were characterized by recurrent *TNFAIP3* mutations, which were less frequent in NMZL. Consistent with these observations, it was recently shown that *TNFAIP3* mutations are very common in ocular adnexal MZL and particularly associated with *IGHV4-34* immunoglobulin gene rearrangements [34].

We further sequenced a cohort of SBCL, U cases to check if consideration of mutational data might give useful information for more specific classification additive to the clinico-pathological features. Based on the predominant occurrence of gene mutations of distinct genes in certain defined lymphoma types (NMZL, ENMZL, SMZL, and LPL), we propose that their detection can aid allocating ambiguous cases into more defined diagnostic categories. For example, in two cases *MYD88* mutations indicated a likely LPL classification. Similarly, *TNFAIP3*, *CREBBP*, *EZH2*, and *TNFRSF14* could predict a more likely NMZL classification in three other cases. Hence, application of targeted panel sequencing can give valuable additional clues for classification of ambiguous SBCL, U cases, in which clinical, morphological, and phenotypical features are equivocal.

Collectively, our study identifies for the first time recurrent *BRAF* mutations in NMZL as well as somatic mutations that could help to discriminate it from other closely related small B-cell lymphomas. Larger-scale prospective studies are still missing to assess the diagnostic accuracy and the predictive value of *BRAF* and other somatic mutations in NMZL.

## Electronic supplementary material

Suppl Figures

Suppl. Table 1

Suppl. Table 2

Suppl. Table 3

Suppl. Table 4

Suppl. Table 5

Suppl. Table 6

Suppl. Table 7

Suppl. Table 8

Suppl. Table 9

Suppl. Table 10

Suppl. Table 11
