# Supplementary material for: High-throughput sequencing of nodal marginal zone lymphomas identifies recurrent BRAF mutations
Source: Leukemia. 2018 Feb 28;32(11):2412–26. doi: 10.1038/s41375-018-0082-4 (PMC6224405; doi:10.1038/s41375-018-0082-4)
Supplement: Supplementary file 1 — Suppl Figures [file 41375_2018_82_MOESM1_ESM.pdf]

High-throughput sequencing of nodal marginal zone lymphomas identifies recurrent *BRAF* mutations

V. Pillonel[1*], D. Juskevicius[1*], C.K.Y. Ng[1, 4], A. Bodmer[2], A. Zettl[3], D. Jucker[1], S. Dirnhofer[1], A. Tzankov[1]✉

[1]Institute of Medical Genetics and Pathology, University Hospital Basel, University of Basel, Basel, Switzerland

[2]Institute of Pathology, Cantonal Hospital Baselland, Liestal, Switzerland

[3]Pathology, Viollier AG, Allschwil, Switzerland

[4]Department of Biomedicine, University of Basel, Basel, Switzerland

[*]contributed equally to this study

✉ Correspondence: Prof. Dr. med. Alexandar Tzankov, Head Histopathology and Autopsy, University Hospital Basel | Pathology, Schoenbeinstrasse 40 | CH-4031 Basel, Tel. +41 61 265 3229 | Fax +41 61 265 3194, E-Mail: alexandar.tzankov@usb.ch

Supplementary Figures

Suppl. Figure 1

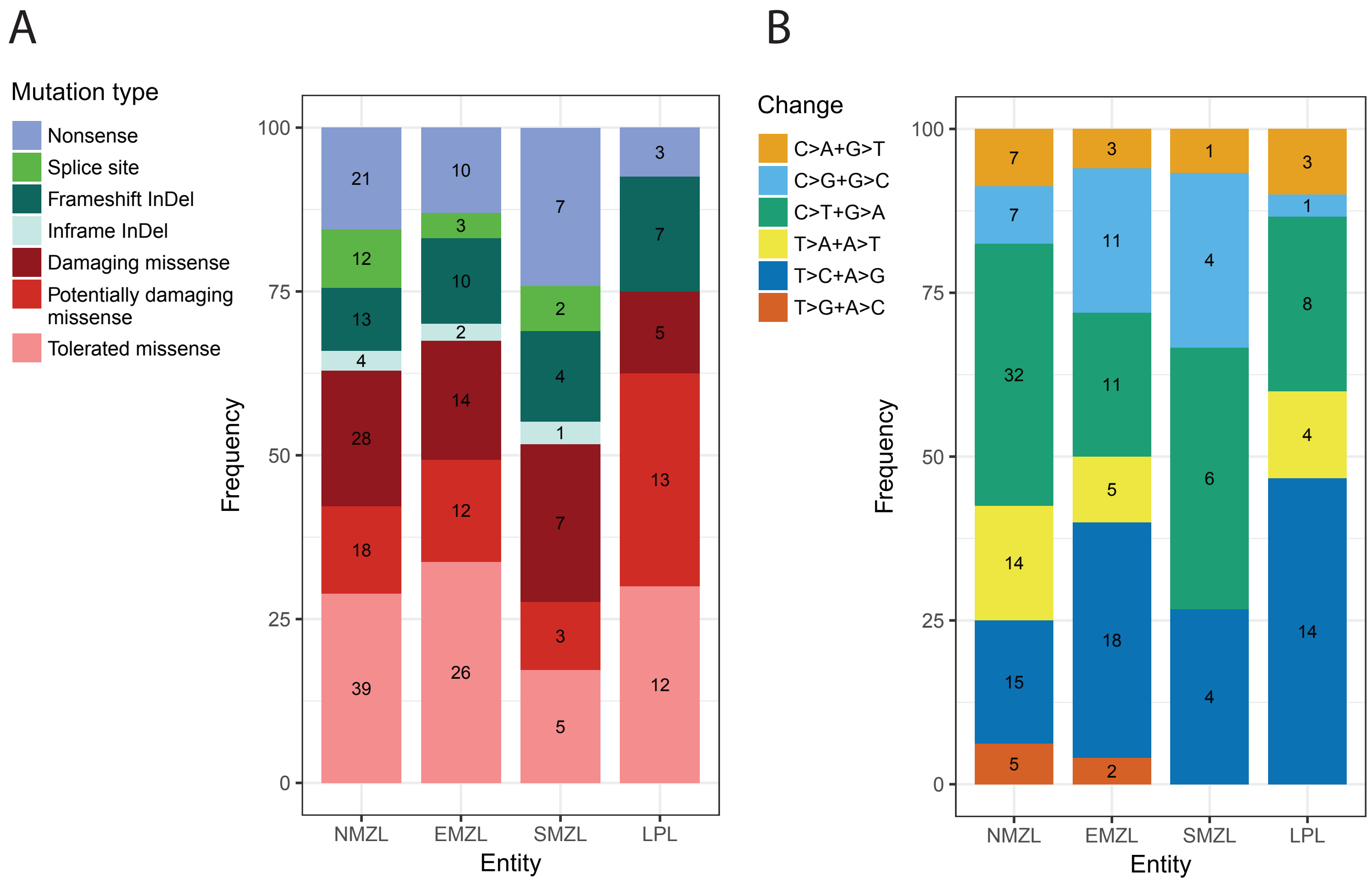

Suppl. Figure 1. Overview of detected mutations by targeted high-througput sequencing.
(A) Number and type of non-silent somatic mutations identified in the 4 small B cell lymphoma cohorts (NMZL n=25, EMZL n=32, SMZL n=12 and LPL n=11). The functional impact of missense point mutations was evaluated with Meta LR algorithm based on rankscore values as follows: 0 ≤ tolerated ≤0.6; 0.6 < potentially damaging ≤ 0.811; 0.811< damaging ≤ 1. (B) The pattern of nucleotide substitutions in the 4 cohorts reveals a predominance of transitions over transversions (ratio ≥1.4 (≥58%), range 1.4-2.75 (58-73%)) in all cohorts.

Suppl. Figure 2

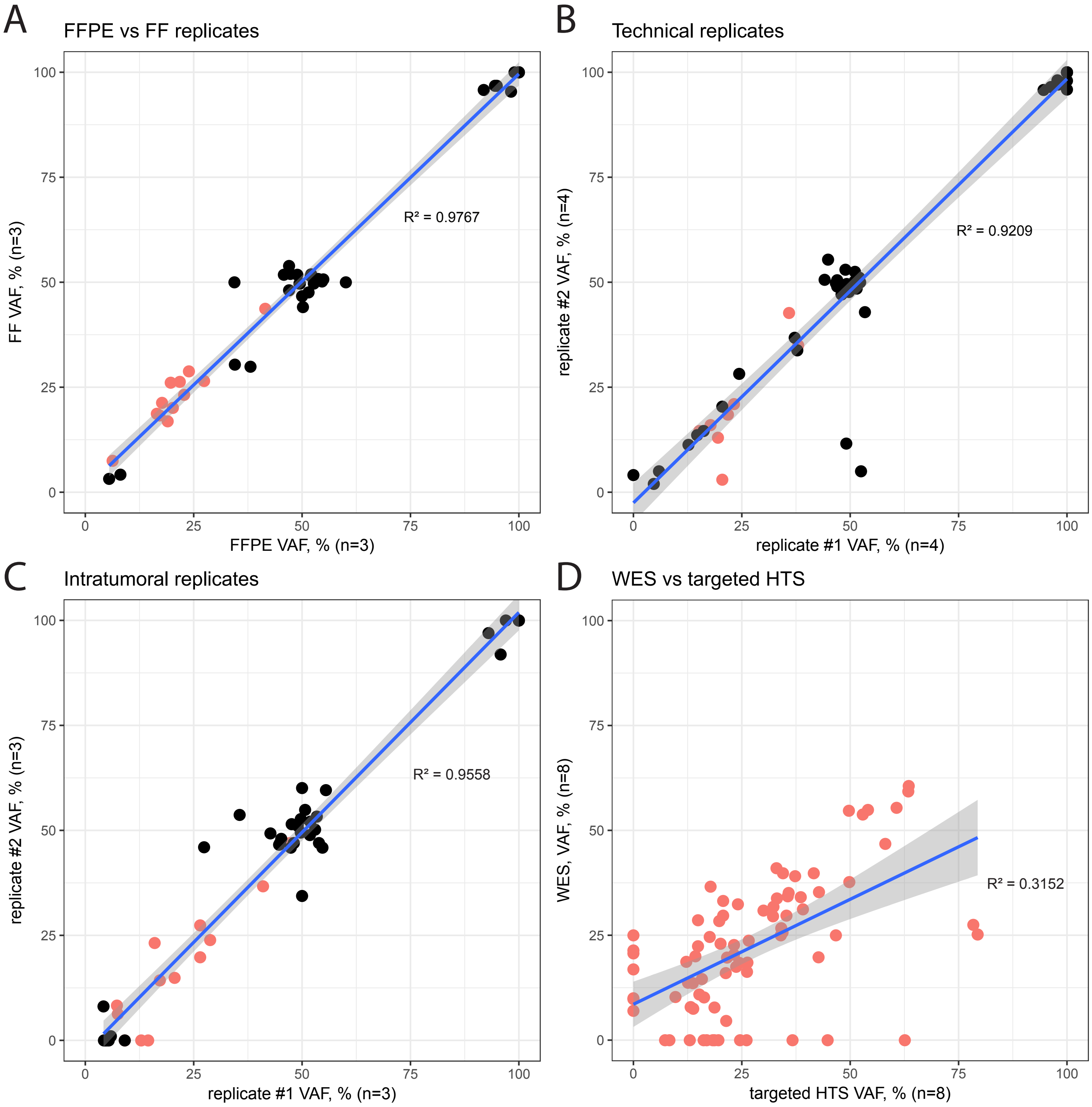

Suppl. Figure 2. Validation of sequencing data.
Sequencing replicates were performed in order to test the robustness of mutation detection. (A) formalin-fixed and paraffin-embedded (FFPE) vs. fresh frozen (FF) replicates (B) technical replicates, (C) intratumoral replicates (DNA isolated from different parts of sampe patient's sample) and (D) whole exome sequencing (WES) vs. targeted high-througput sequencing (HTS) replicates Plotted are variant allelic frequencies (VAF) of mutations detected by each repetitive sequencing run. VAF of 0% indicates that a respective mutation was undetected in one of the samples and represents either a false-positive or a false-negative variant call. Germline and synonymous somatic mutations are shown in black, non-synonymus somatic mutations in red. Linear regression is shown with 95% confidence interval (shadow).

Suppl. Figure 3

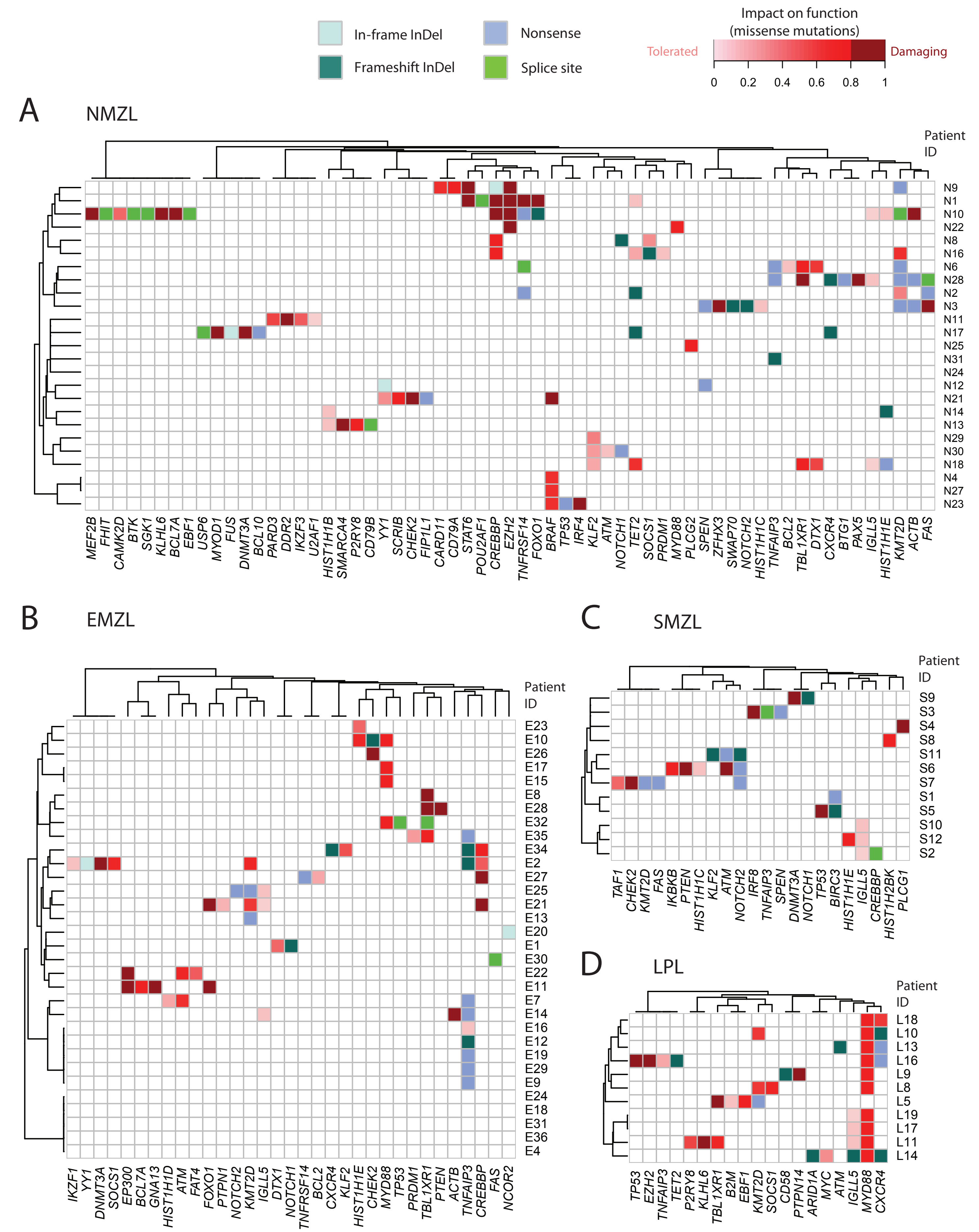

Suppl. Figure 3. Somatic mutations in small B cell lymphomas clusterd by genes and samples.
Heatmap plot showing the distribution of all non-synonymous mutations detected by high-througput sequencing in all four small B cell lymphoma cohorts: (A) nodal marginal zone lymphoma (NMZL); (B) extranodal marginal zone lymphoma (EMZL); (C) splenic marginal zone lymphoma (SMZL); (D) lympho-plasmacytic lymphoma (LPL). The heatmap is hierarchically clusterd by genes and samples with each row representing a primary tumor and each column representing a gene.

Suppl. Figure 4

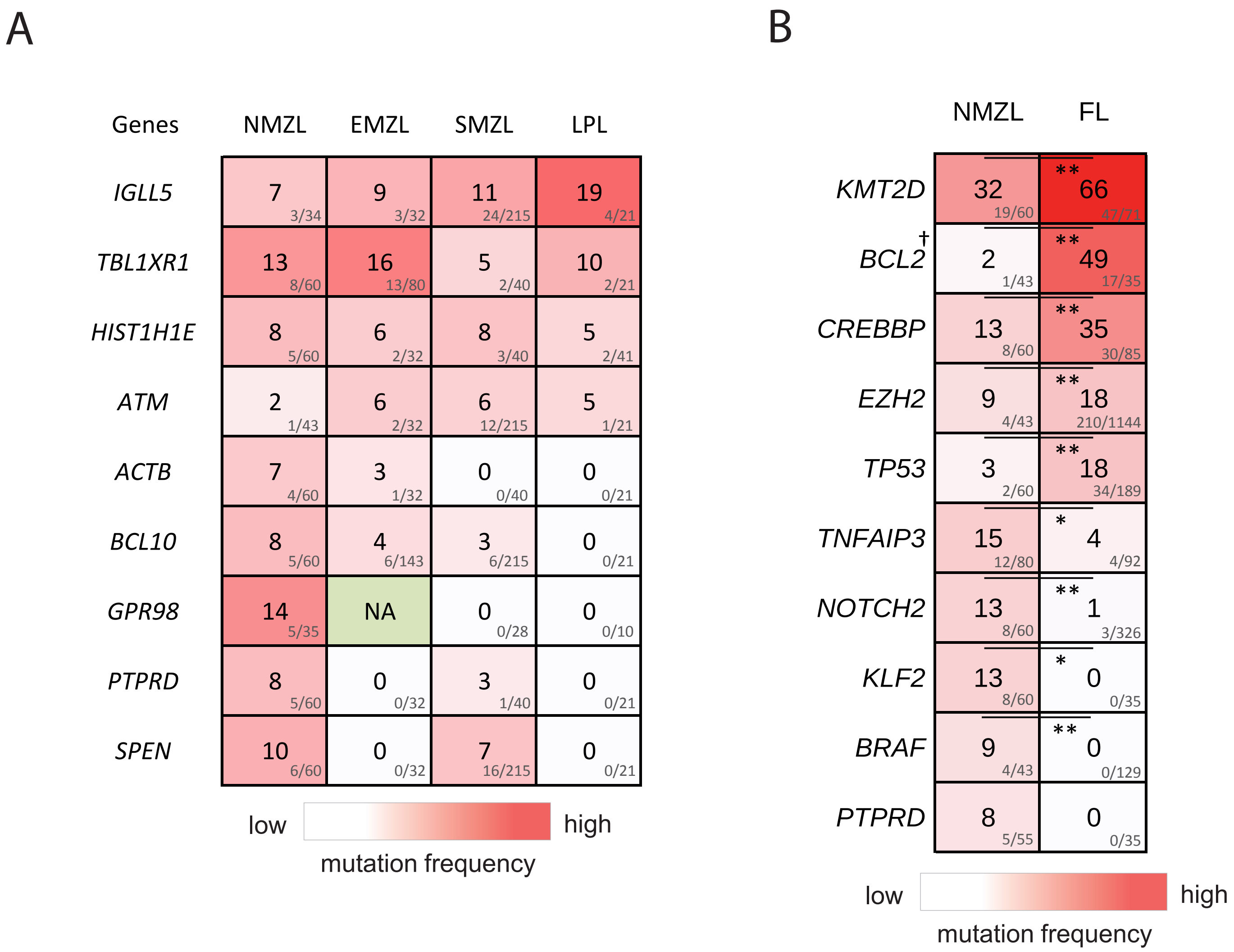

Suppl. Figure 4. (A) Meta-analysis results between SBCL for additional genes, which did not reach statistical significance. p-value was calculated with the Fisher's exact test. (B) Comparison of gene mutation frequencies between aggregated NMZL dataset and FL dataset downloaded from COSMIC. Only genes with statistical significance by two-way Fisher's exact test are shown. NA: not available data sets. *: p≤0.05; **: p≤0.01; † - only BCL2 point mutations are considered in this comparison, excluding t(14;18).

Suppl. Figure 5

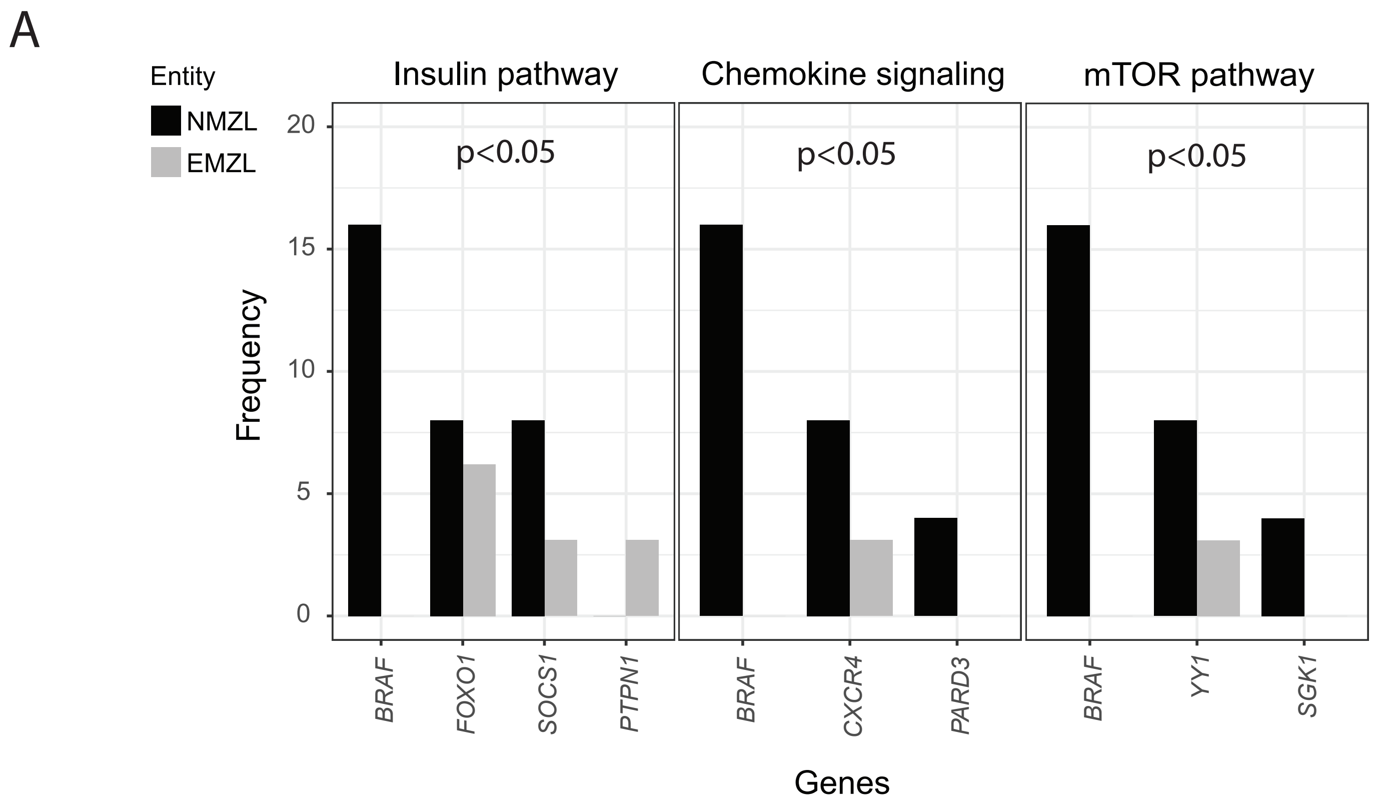

Suppl. Figure 5. Differentially affected pathways in nodal marginal zone lymphoma (NMZL) vs. extranodal marginal zone lymphoma (EMZL).
Differentially mutated pathways between NMZL and EMZL are shown. Affected genes in each patways is depicted by frequency. Statistical significance as assessed by the Fisher's exact test is shown for each pathway.
